# Barriers and aids to routine neuromuscular monitoring and consistent reversal practice—A qualitative study

**DOI:** 10.1111/aas.13606

**Published:** 2020-05-06

**Authors:** Jakob L. D. Thomsen, Adrian P. Marty, Shin Wakatsuki, Alex Macario, Pedro Tanaka, Mona R. Gätke, Doris Østergaard

**Affiliations:** ^1^ Department of Anaesthesiology Herlev Hospital Copenhagen Denmark; ^2^ Department of Anaesthesiology Stanford School of Medicine Stanford CA USA; ^3^ Copenhagen Academy for Medical Education and Simulation and University of Copenhagen Copenhagen Denmark

**Keywords:** focus group interview, neuromuscular blocking agent, neuromuscular monitoring, qualitative methods

## Abstract

**Background:**

Neuromuscular monitoring is recommended whenever a neuromuscular blocking agent is administered, but surveys have demonstrated inconsistent monitoring practices. Using qualitative methods, we aimed to explore barriers and aids to routine neuromuscular monitoring and consistent reversal practice.

**Methods:**

Focus group interviews were conducted to obtain insights into the thoughts and attitudes of individual anaesthetists, as well as the influence of colleagues and department culture. Interviews were conducted at five Danish and one US hospital. Data were analysed using template analysis.

**Results:**

Danish anaesthetists used *objective* neuromuscular monitoring when administering a non‐depolarizing relaxant, but had challenges with calibrating the monitor and sometimes interpreting measurements. Residents from the US institution used *subjective* neuromuscular monitoring, *objective* neuromuscular monitoring was generally not available and most had not used it. Danish anaesthetists used neuromuscular monitoring to assess readiness for extubation, whereas US residents used subjective neuromuscular monitoring, clinical tests like 5‐second head lift and ventilatory parameters. The residents described a lack of consensus between senior anaesthesiologists in reversal practice and monitoring use. Barriers to consistent and correct neuromuscular monitoring identified included unreliable equipment, time pressure, need for training, misconceptions about pharmacokinetics of neuromuscular blocking agents and residual block, lack of standards and guidelines and departmental culture.

**Conclusion:**

Using qualitative methods, we found that though Danish anaesthetists generally apply objective neuromuscular monitoring routinely and residents at the US institution often apply subjective neuromuscular monitoring, barriers to consistent and correct use still exist.

**Editorial Comment**

Inadequate monitoring of neuromuscular blockade and reversal of neuromuscular blocker drug effects can contribute to preventable perioperative patient complications. This qualitative assessment of specialty physician approaches to these issues in two high‐income countries and practices show that these issues remain an area where more education and better implementation of the best practice standards can be needed.

## 1 INTRODUCTION

The degree of paralysis after administration of neuromuscular blocking agents (NMBA) can be assessed using a peripheral nerve stimulator in *subjective* neuromuscular monitoring, or with an *objective* (quantitative) neuromuscular monitoring device, for example by means of acceleromyography.^1^ In a recent consensus statement, researchers in the field recommended that objective neuromuscular monitoring should be used whenever a non‐depolarizing NMBA is administered to reduce morbidity associated with residual neuromuscular block.^2^ The importance of also monitoring the depolarizing block after succinylcholine was demonstrated in a study of awareness at emergence in patients with butyrylcholinesterase (BChE) deficiency.^3^ However, surveys and observational studies show that often, objective neuromuscular monitoring equipment is either not available or used inconsistently.^4^, ^5^, ^6^, ^7^, ^8^, ^9^ Naguib and colleagues found that most anaesthesiologists in the United States (US) have access to *subjective* neuromuscular monitoring, but few had access to *objective* neuromuscular monitoring compared to their European colleagues.^10^ The survey also showed that even when both objective and subjective monitoring were available, US respondents were more likely to use the subjective method, though it did not address why. Furthermore, routine reversal of the neuromuscular block was less common among European respondents.^10^ Already in 2005, most clinicians in Denmark had access to objective neuromuscular monitoring.^11^ However, a more recent survey found that 75% of Danish anaesthetists had technical difficulties with the objective monitors at least 25% of the time.^12^ While the surveys have demonstrated a large variability in availability and use of neuromuscular monitoring equipment, little is known about potential barriers to anaesthetists’ routine use of neuromuscular monitoring.

Using qualitative methods, we aimed to explore barriers and aids to routine neuromuscular monitoring and consistent reversal practice.

## 2 MATERIALS AND METHODS

We conducted a qualitative study based on focus group interviews and surveys of anaesthesiologists’ and nurse anaesthetists’ clinical practice and used systematic text condensation to extract themes as described by Malterud.^13^

### 2.1 Participants

Focus group interviews were conducted in Denmark between August 2014 and January 2015. When author JLDT was a visiting scholar at the Department of Anaesthesiology at Stanford Medical Center in the Spring of 2017, interviews were conducted with anaesthesia residents to explore differences and similarities between an institution that had not yet implemented objective neuromuscular monitoring and the previously obtained findings from Danish hospitals. In Denmark, we recruited both smaller and larger departments. For the focus group interviews, we included certified and in‐training anaesthesiologists and nurse anaesthetists (collectively referred to as ‘anaesthetists’).Participants were incentivized to stay after normal work hours with a gift certificate (approximate value of €30), resulting in a convenience‐based sample. In the US, we included anaesthesia residents from the Department of Anaesthesiology at Stanford Medical Center. Three specific lectures were allotted as time slots for interviews with first, second‐ and third‐year residents, respectively, resulting in a convenience‐based sample. Secondly, we sent a survey to the attendings at the department and other anaesthesia residency programmes in California, US.

### 2.2 Focus group interviews

We chose focus group interviews as the primary approach because group interaction would encourage respondents to explore and clarify individual and shared perspectives.^14^ An interview guide (Appendix 1) was developed based on literature review and the authors’ expertise in the subject. Participants were informed that the purpose of the study was to get their view on neuromuscular monitoring and describe the challenges they experience with the subject. They were informed about potential conflicts of interest. The author, JLDT, who had experience from a previous interview study,^3^ conducted the interviews as a research fellow in Denmark, and as a visiting scholar at the US institution. In Denmark, additional interviews were conducted until data saturation was achieved as judged by author JLDT, whereas in the US, the interviews were limited to residents at a single programme. The interviews were recorded on a voice recorder and transcribed in their entirety by a research assistant or author JLDT.

### 2.3 Survey of attendings

We designed an online survey to get the views of attending anaesthesiologists in the US institution on the subjects discussed by residents. The survey contained a total of 14 multiple‐choice and open‐ended questions. The survey was sent to attendings at the local institution and to two attendings at each of the ten other anaesthesia residency programmes in California.

### 2.4 Ethics committee approval

According to Danish regulations, approval from the local ethical committee is not required for this type of studies. The Danish part of the study was registered at clinicaltrials.gov (NCT02239965). The Institutional Review Board at Stanford University approved the study protocol for the US part of the study (Protocol # 42091). Verbal consent was obtained from all participants.

### 2.5 Analysis

Initially, the author JLDT read the transcripts freely, without coding or analysing. The transcript of one focus group interview was then coded, using a priori codes based on the interview guide and own expertise in the subject. Further codes were derived directly from the text as a part of the analysis. Initial coding of the Danish interviews was discussed and revised by the authors JLDT and DØ. The resulting codebook was used as a starting point for the coding of the remaining interviews. Finally, the survey responses were coded. All codes from both Danish and US data were plotted in concept maps to group related codes into themes and subthemes and demonstrate relations.^15^ Quotes reported in the results were cleaned for slang phrases and condensed. Danish quotes were translated by author JLDT.

Findings are reported according to the Consolidated Criteria for Reporting Qualitative Research (COREQ) where appropriate.^14^ Coding was performed in nVivo 11 for Mac (QSR international).

## 3 RESULTS

We conducted 6 focus group interviews in 5 Danish teaching hospitals, with five to seven participants in each interview, totalling 40 interviewees. In the US, we conducted three interviews with a total of 21 anaesthesia residents. Surveys were completed by 22 local attendings and by 13 attendings from other programmes in California. The duration of the focus group interviews was 37‐65 minutes. Analysis of the interviews resulted in three major themes: *Use of neuromuscular monitoring and reversal* and *Barriers and aids to routine neuromuscular monitoring*, and *Neuromuscular monitoring after* succinylcholine. Table 1 and Appendix 2 show the themes with subthemes, codes and illustrative quotes.

**TABLE 1:** Barriers and aids to routine neuromuscular monitoring

Subtheme	Code	Source	Quote or observation
Barriers	Residual block unrecognized	DK‐1	But the others, where it is not discovered until the patient cannot cough sufficiently, and so on, and gets pneumonia, they are probably underreported.
	Residual block unrecognized	US‐3	I think there are patients with residual block in the PACU and we may clinically by vital signs not notice or it may not be obvious to us.
	Residual block unrecognized	US‐3	Well, I think that we have heard many patients say that they're a little weak or groggy or whatever—how much of that is just our hand waving ‘oh that's normal after surgery and anaesthesia’ vs what is residual block? I don't think I can say, confidently.
	Residual block unrecognized	US‐1	It was an obese woman in the PACU, saturating 75%, blue. And we gave 400 of sugammadex and she was totally fine, and I thought ‘oh, that does happen’. [Attending] was there and she said ‘look, she's kind of twitching, this is what residual neuromuscular block looks like’. Because I said ‘let's give flumazenil too’ and she said ‘no, let's just give sugammadex and see what happens’. And she perked right up.
	Equipment placement	DK‐5	We remove [the cable of the monitor] and put in the drawer when it is not in use. I actually managed to take the cable from the drawer, and put it on the patient without plugging it into the monitor. It took me a while to find the source of the error.
	Equipment placement	US‐2	The twitch monitors that they do have, some of them are tethered to the anaesthesia machine, so either it doesn't reach and you have to pull your machine closer or it dangles in your way and gets tangled in your lines and it's just an inconvenience or it doesn't work, the battery's out.
	Reliability	US‐3	And the problem with the questionable working [peripheral nerve stimulators] is you don't know if it's not working or if you don't have twitches… (...) often times you'll get another one and then that one doesn't work either—or do I not have twitches?
	Reliability	DK‐3	I always use neuromuscular monitoring when using non‐depolarizing blockers, but I do not trust it completely because it always calibrates to more than 100%
	Reliability	DK‐2	That is probably also why many anaesthesiologists, myself included, get tired of the equipment when it does not work: the patient is awake and breathing fine, and the TOF monitor says ‘0’. You get furious and tear it off. Then the problem arises when it says ‘0’ and the patient is actually paralysed, but you still tear it off and throw it away and awaken them. That would be a mistake, of course, but that is because we do not have equipment that is 100% reliable and you become insecure about its performance, and was it calibrated or not? and can you count on it?
	Reliability	US‐2	A lot of time you can't get a good signal and you cannot get [the quantitative monitor] to go correctly and then you just ignore it.
	Time pressure	US‐2	…if it wasn't like 11 pm at night and there wasn't like this production pressure to get it done, we should have stayed in the OR for fifteen minutes, while it was like ‘oh it's okay, it'll be fine’ and then we had to re‐intubate in the PACU and wait for forty minutes.
	Time pressure	US‐3	I still probably wouldn't use it because you have to do it before you give paralysis, but we usually are in such a rush, once you start sedating you're going to give the paralysis. I don't have time to put on another monitor, give sedation, check, calibrate the accelerometer and then give paralysis and intubate. It's too much of a hassle for too little gain.
	Arms tucked	DK‐2	There is often the challenge with one arm being tucked down the sides, and you have to put two venous catheters, an arterial canula, a TOF monitor, and a pulse oximeter, and it all goes into that one arm.
	Arms tucked	US‐2	[With the arms tucked, the objective monitor] is not reading correctly, so you have to get their hand like correctly and clearly they have a good strong twitch, but because of the way their thumb is going down it is not reading and so, it becomes useless to me.
Attitude	Necessity	DK‐2	If you are doing a procedure where you only need paralysis for intubation and the procedure is less than an hour or so, I think monitoring of the block is quote‐unquote redundant, because of what we know about pharmacokinetics and so on, it should be eliminated long before we awaken the patient—using those 15‐20 mg doses [of rocuronium]
	Necessity	US‐3	The IVs and art lines are in the way, I feel like it's more trouble than benefit, unless there's a specific indication that I want to like very closely objectively monitor paralysis, but that would be so rare I wouldn't use it regularly
	Necessity	US‐1	Well, I think there is just a sense of that we get a good enough sense of where we're at [with subjective monitoring]. Maybe people are a little more cavalier too, with having sugammadex because you always have something to fall back on if any problems were to occur.
	Necessity	US‐1	I know that the quantitative machine can tell you a lot more, but I’ve yet to encounter an instance where someone was still weak based on the non‐quantitative approach
	Significance of residual block	US‐3	How clinically significant is a minor residual paralysis in most patients if they're able to cough, but they're still a little weak? [other residents agreeing: ‘yeah’ and ‘right’] They're going to be in the PACU sort of groggy for an hour anyway, not knowing, not remembering (…) we probably have patients who have residual paralysis, but for a lot of them it doesn't become clinically relevant.
	Significance of residual block	DK‐1	Well, it is a serious matter if they cannot breathe when you pull the tube if you did not realize it. Very much so, psychologically, but it is also dangerous if re‐intubation is difficult and you cannot get them sedated deeply enough. So, it would certainly be a problem, if it were something we experienced often.
	Experience with residual block	US‐3	In the PACU, where I saw a lot of patients having residual paralysis, where the intraoperative team was convinced that they had been reversed, also sort of changed my practice of making sure you are checking before and after you have given reversal. Because often times, they haven't checked after.
	Experience with residual block	US‐2	I have had it happen twice. (…) and it was also like a fairly short case and I think my attending said ‘yeah, it should be okay, I think it'll be fine’. We gave a full dose [of neostigmine] and then, in the PACU she was in respiratory distress and had to be re‐intubated, she was hypoxic, 91%.
	Experience with residual block	DK‐1	I do not know how many years back it was since we had someone with severe residual neuromuscular block, but it must be some years back, because I am in the patient safety group, and I do not recall that there has been a case in the last 3‐4 years.
	Experience with residual block	US‐2	Objectively it seemed okay, but then as soon as we got to PACU, we had to reintubate and sit with the patient for half an hour. So, you can get fooled even if they're bucking.
	Attending consensus	US‐1	It does feel a little bit like the wild, wild west where people just kind of do what they want. (…) individual practice vary so widely which is really frustrating as a trainee, cause you're just like ‘what am I supposed to do? What do you want me to give?’ and it really varies from attending to attending
	Attending attitude	US‐1	Part of the challenge too is the papers that looked [at residual block] will often talk about how they don't even checked twitches. And so, I think, when I talk to attendings about this they sort of spin it like ‘well that was just gross negligence and certainly doesn't apply to my practice’.
	Attending attitude	US‐3	They had given reversal and the patient had a prolonged wake‐up, and the patient was large, had obstructive sleep apnoea and had had thoracic surgery and wasn't taking great breaths and was obstructing and it seemed like the anaesthetic had worn off. So, we got a twitch monitor and I didn't see great twitches. I talked to the attending and said: ‘I’m going to give sugammadex reversal’, the attending said ‘no, the patient was fully reversed, I don't agree’. Then I couldn't really do anything, I kept on checking and then I just gave [sugammadex] and then they were fine.
	Attending attitude	US‐3	It would have to be something that we've been taught as a first‐year resident and on. And it has to be uniform by the attendings, it can't be like some attendings saying ‘don't worry about it’. It's like when they want us to start using the sterile kit for the arterial line, some attendings say ‘yes, use it’, some attendings say ‘I don't care’. If you offer the lazier thing as okay, then I’m going to take the lazier one—unless there is some important indication to do the harder work.
	Attending attitude	US‐3	If we want to use [objective neuromuscular monitoring] at the [other hospital], then the attendings will be fine with it, but sometimes the attendings want to use it then we use it. Sometimes, attendings don't care.
Department	Equipment availability	US	[On *subjective* neuromuscular monitoring] It is not always immediately available in every room
	Equipment availability	DK‐1	We have the *objective* neuromuscular monitoring module in all our machines
		DK‐5	It is more readily available now, because before, we were running around, asking where the monitor was. Even though we had 4 or 5 it felt like they were always missing.
	Equipment availability	DK‐1	In [one department], we have to go look for them, whereas in [other department], it is available in the operating rooms.
	Availability/experience	US‐1	At [other hospital], I think they have a hookup to actually do quantitative, never seen it used I’ve used it once at the [other hospital] I’ve never used it. I never even knew we had it, I had no idea that was an option.
	Culture	US‐1	Where I went to medical school and did a good amount of anaesthesia, we always hooked up to the larger monitor where you actually get a quantitative value of your train‐of‐four, it was just one of the monitors that you put on at the beginning of the anaesthesia every time, just like blood pressure or EKG. I found it very odd when I arrived here and no one did and we seem to use the twitch monitor in a heterogeneous way, it was very different from attending to attending. I guess I have adapted now, but at first it seemed strange.
	Culture	DK‐1	I perfectly understand that, if you come from a place where you are used to routinely apply monitoring in all cases, and then you are at a new place and you are constantly questioned about why you are doing it and they do not do it here, and then you sort of go ‘well, okay then’.
	Culture	DK‐1	I had my first year of residency at [other hospital] where if you did not redose rocuronium, you would not monitor the block and you would awaken the patient after an hour and test for residual block by having the patient lift their arm.
	Culture	US‐2	I mean I think the culture here is that you know prolonged paralysis or re‐curarization after reversal is like really dangerous and it's like a safety concern
	Standardization	US‐1	Just standardization of practice is helpful as well, especially with like neostigmine, because it seems it was all over the place
	Practice vs guidelines	DK‐4	It may be a bit self‐contradictory, but I did write in the standard operating procedure, that you have to [use monitoring] if you give a NMBA. But I have to be honest and confess that if I used succinylcholine for a rapid sequence induction, I will rarely put a TOF monitor on.
Aids	Integration of NM	DK‐5	Because you see it much more frequently. You are staring at the [monitor] all the time anyway. Instead of going ‘oh, I have to check the TOF monitor, too. Oh, my—32%,’! Now you see it all the time, and check everything on the screen. You look up, and then your eyes naturally pass the lower right corner.
	Routine	DK‐2	I hardly think we have any patients who receive rocuronium without being monitored
	Legal consequences	US‐1	Many attendings have been like ‘this is an easy box to check if there is a claim into this case—if there's any question as to whether you assessed neuromuscular block, could you defend this in court?’ Which is not a good reason to do anything in this country, but it is a part our allegiance
	Reliability	DK‐1	A condition for wanting to use any equipment is simply knowing that it works every time, which is true for the new monitor.
Need for training	Setup	US (S)	[I would like training in] how to place a quantitative device so that data are reliable
	Setup	US‐2	[I would like] being able to assess the twitches on the hands, even when they're tugged, instead of just being lazy and putting it on the head
	Request training	US‐3	[I would like] training in [objective neuromuscular monitoring] so we know how to use it instead of just having it available
	Calibration	DK‐5	If it will not calibrate, that is a situation where I wonder ‘how does it actually affect the measurements’?
	Calibration	US‐3	You can apply it [accelerometry without calibration], it's just not calibrated so the usefulness of it, how objective it really is, is also in question and it's harder to interpret.
	Troubleshooting	DK‐5	I don't understand why it always calibrates to 1.14 or something else above 1.0
	Risk stratification	US‐1	[What are] the patient populations that are truly going to be affected by this? Who is not reversed adequately? Who is super sensitive to non‐depolarizing? Because if you have a lot of these healthy patients where you give it and it doesn't do anything to change their outcome, or you don't give it and it doesn't do matter. And then your sick patients for whom you could really make a difference in your quality of care.
NM after succinylcholine	No pain	DK‐3	Let us say, then, that you had an awareness experience, and you were lying there after a short procedure—you are not having surgery at that time, so there is no pain. There is the feeling of suffocation, that is certainly not comfortable, either, but it is not major heart surgery or emergency caesarean section.
	BChE rare	DK‐1	There are many things we see once, or has happened in the last 10 years at this hospital, but that does not necessarily lead to a bigger change, because then you would have to change things all the time, which perhaps induces uncertainty because people are not confident in what they are doing.
	BChE deficiency often known	DK‐1	But it is often caught in the pre‐anaesthetic assessment if someone in the family had BChE deficiency
	Will be discovered eventually	US‐2	No, I think probably if at the end of the case you run into trouble and somebody looks weak, then you might do it, but not routinely.
	Will be discovered eventually	US‐3	I mean I guess you checked twitches once you found out, right? So, whether you checked at the beginning of the case or at the end of a case it wouldn't have really made a difference.
	Will be discovered eventually	US‐1	I put them on pressure support at the end and see if they breathe. If they didn't then I’d go down that pathway.
	Over‐complicating	DK‐1	It is also a question of not making a relatively simple procedure into a big thing. As soon as you start putting more wires on the patient and doing stuff, then maybe they need a central venous catheter, too, and then they all get an arterial canula. How intensely should a perfectly healthy patient having a 15‐minute gastroscopy be monitored?
	Over‐complicating	DK‐3	I also think that sometimes with the short procedures, we spent some time [applying monitoring] and resources that could have been spent differently, because you know what the half‐life is. And if there is a problem, you put the monitor on.
	Time pressure	DK‐4	You should not underestimate the effect of this being a production, and it has to run smoothly, and we should not prolong patient turnover times
	Experience changing behaviour	US‐3	And then I usually always check at least once after I’ve given it to put in document, but that's because I’ve had a case where I gave it and then four hours later didn't check it prior to wean somebody of the ventilator and then they were still paralysed and I realized that they had pseudocholinesterase deficiency. So now I check everybody
	Experience *not* changing behaviour	DK‐1	Well, we do have a colleague who was anaesthetized and she had [BChE deficiency]. And she did not think it was particularly funny to wake up, and get succinylcholine, and not being able to breathe, so… Maybe we should consider it, but we have given it so many times without.
	Experience	US‐3	It was during my PACU rotation, patient just didn't move after surgery, so we brought her out on the ventilator and they had to extubate her like a couple of hours later. And we thought that it was most likely pseudocholinesterase deficiency and she hadn't mentioned anything but she had had surgery years before and was like ‘oh, yeah it took me a while to wake up’.
	Routine	DK‐5	I never used the monitor for rapid sequence induction, did you? If only using succinylcholine? I never did that. I used to do it in the old days, but now, I mostly do not
	Before non‐dep.	DK‐3	But it does not have to be on for intubation with succinylcholine, but it is applied and turned on before administering rocuronium.
	Before non‐dep.	US‐1	And I try to remember to always check it if I gave succinylcholine before I give rocuronium to make sure that the twitches came back.

*Note* Themes, subthemes, codes and illustrative quotes from focus group interviews and surveys in Denmark (DK) and the institution in the United States (US). The number after ‘DK’ indicates department number, whereas the number after ‘US’ indicates year of residency for the interviewee. ‘S’ indicates data from surveys.

Abbreviations: BChE, butyrylcholinesterase; NMBA, neuromuscular blocking agent, TOF, Train‐of‐four.

### 3.1 Theme 1: use of neuromuscular monitoring and reversal after non‐depolarizing NMBA

**FIGURE 1:**
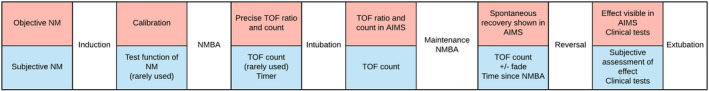
Typical management of neuromuscular block, depending on type of equipment available. Red: objective neuromuscular monitoring, blue: subjective neuromuscular monitoring (twitch monitor). AIMS, anaesthesia information management system; NM, neuromuscular monitoring; NMBA, neuromuscular blocking agent; TOF, train‐of‐four [Colour figure can be viewed at wileyonlinelibrary.com]

The Danish and US anaesthetists generally had access to different types of neuromuscular monitoring. Neuromuscular block was managed differently, depending on the equipment available, as shown in Figure 1. Danish anaesthetists stated that they always apply acceleromyography before administering the non‐depolarizing NMBA to check the control response, calibrate the monitor and guide timing of intubation. They would use the monitor to guide maintenance dosing of NMBA, timing and dosing of reversal, and readiness for extubation. The residents at the US institution had access to primarily subjective neuromuscular monitoring and would rarely apply it before administering the first dose of NMBA, but consistently apply it to guide maintenance dosing of NMBA and timing of reversal. They did not check a baseline response before administering NMBA, which sometimes led to confusion about whether the monitor was malfunctioning or the patient was still paralysed when no responses were observed. Residents often used a timer to assess readiness for tracheal intubation, stating 2 minutes as the usual time from administration of NMBA to intubation.

When reversing the neuromuscular blockade, Danish anaesthetists would give a standardized non‐weight‐based dose of one vial (neostigmine 2.5 mg and glycopyrronium 0.5 mg) and then assess the effect with acceleromyography. They would then rely on the monitor for assessing readiness for extubation, often combined with clinical tests. The residents at the US institution and the attendings based their decision to administer reversal on a range of parameters: duration of the case, time since last administration of NMBA (from 2 to 6 hours) and subjective neuromuscular monitoring. Reversal dosing was either based on subjective monitoring or the same standard weight‐based dose was given to all patients. Some residents would always administer at least a small dose of neostigmine, referred to as a ‘legal reversal’. Some residents used train‐of‐four (TOF) and other stimulation patterns to assess readiness for extubation, whereas others would use ventilatory parameters or other clinical tests instead.

### 3.2 Theme 2: barriers and aids to routine neuromuscular monitoring

**FIGURE 2:**
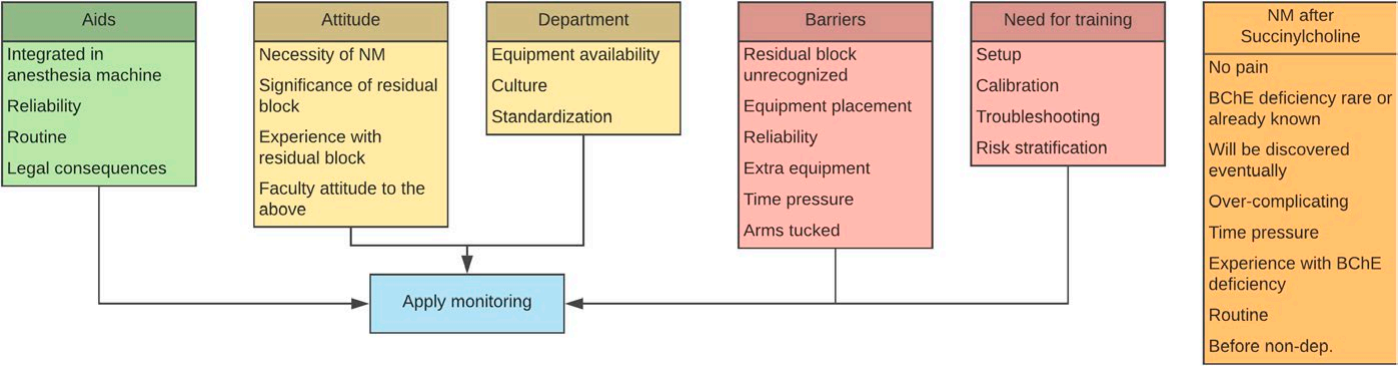
Barriers and aids to neuromuscular monitoring. ‘Barriers’ and ‘Aids’ are shown in red and green, respectively, whereas ‘Attitude’ and ‘Department’ are shown in yellow because they can influence behaviour in either way. The orange box shows factors specifically influencing monitoring behaviour when succinylcholine is administered. BChE, butyrylcholinesterase; non‐dep, non‐depolarizing [Colour figure can be viewed at wileyonlinelibrary.com]

Based on the interview data, we identified barriers and aids to routine neuromuscular monitoring. As shown in Figure 2, we further found that attitudes and departmental factors could influence monitoring practice both negatively and positively, that is act both as barriers and aids.

#### 3.2.1 Barriers

Though monitoring was readily available, even just storing the cable of the monitor in a drawer could pose a barrier to use of the equipment. Residents from the US institution mentioned faulty twitch monitors and flat batteries, and anaesthetists from both countries reported unreliable equipment leading to not trusting its measurements. Time pressure was also given as an explanation for not monitoring and the patients having arms tucked under surgical drapes and not easily accessible was another cause for frustration. There was a need for training in the setup and interpretation of objective neuromuscular monitoring, the purpose of calibration and risk stratification of residual block vs side effects from neostigmine reversal, knowing the limitations of subjective monitoring, and, in Denmark, managing acceleromyography showing TOF ratios greater than 100%. Several local attendings also requested training in objective neuromuscular monitoring.

#### 3.2.2 Attitude

The attitude of both the anaesthetist and the attending in charge of patient care was found to influence whether monitoring was applied. While both Danish and US anaesthetists considered residual neuromuscular block a serious complication and stated that minor degrees of residual block were probably underdiagnosed, some residents at the US institution did express that minor degrees of residual block would not necessarily be clinically relevant. Some residents could not imagine routine application of objective neuromuscular monitoring being worth the effort. Some Danish anaesthetists would omit neuromuscular monitoring only if a reduced dose of 15‐20 mg rocuronium was used. Several residents at the US institution had experienced cases with severe residual neuromuscular block, sometimes requiring tracheal re‐intubation, whereas only a few Danish anaesthetists had experienced severe residual neuromuscular block. Senior Danish anaesthesiologists described cases from years prior, when the long‐acting NMBA pancuronium was used routinely. Personal experiences with patients with residual block sometimes led to a change in the anaesthetist´s behaviour with regard to neuromuscular monitoring, but not always. Both Danish and US anaesthetists described instances where senior colleagues would not acknowledge that the patient had residual neuromuscular block. The residents expressed the importance of consensus between the attendings regarding the necessity and practice of neuromuscular monitoring and reversal. A resident noted that some attendings were perhaps reluctant to acknowledge that studies of residual block applied to their own practice.

#### 3.2.3 Department

In Denmark, objective acceleromyographic neuromuscular monitoring equipment was available in almost every operating room and integrated in the anaesthesia machine. At the US institution, residents had access to subjective neuromuscular monitoring either in every room or upon request. Objective neuromuscular monitoring was only available at one centre where residents rotated through in their training, but only few had experience using it. The surveyed attendings at other institutions reported a similar pattern of having subjective neuromuscular monitoring available either in every operating room or upon request, with objective neuromuscular monitoring available at a single site, upon request, or not at all. Both Danish and US anaesthetists discussed how department culture can influence monitoring behaviour:monitoring routines from a former workplace could be ‘unlearned’ when changing to a department with a different monitoring culture. The lack of a standard approach or a guideline regarding reversal was a cause for frustration among some residents, but on the other hand, a Danish anaesthetist reported not applying neuromuscular monitoring after succinylcholine even though he himself had written the guideline mandating monitoring.

#### 3.2.4 Aids

Danish anaesthetists described it as helpful to have the TOF measurements shown directly on the monitoring screen. Both Danish and US anaesthetists discussed the importance of having reliable equipment, and most Danish anaesthetists were of the opinion that the current integrated equipment was better than the stand‐alone monitoring devices that had been available before. Some residents from the US institution described how they applied monitoring solely so they could document it in case a claim was filed.

### 3.3 Theme 3: neuromuscular monitoring after succinylcholine

While some barriers, like time‐pressure and ‘keeping it simple’, also affected application of neuromuscular monitoring after succinylcholine, some arguments against routine monitoring were unique. Opinions expressed included that BChE deficiency is so rare, that if paralysed at the end of anaesthesia the patient would not feel pain, that patients with BChE deficiency will often know beforehand and warn the anaesthetist, or that the deficiency will be discovered eventually, anyway, by the patient not breathing. In some instances, a single experience with severe residual block after succinylcholine was enough to make the anaesthetist apply monitoring routinely, whereas at one Danish hospital, most anaesthetists did not apply routine monitoring despite having a colleague who had severe BChE deficiency and had shared her story of being awake while paralysed.

## 4 DISCUSSION

### 4.1 Use of neuromuscular monitoring and reversal

The US residents mostly used a timer to assess readiness for intubation, whereas the Danish anaesthetist relied on objective neuromuscular monitoring, some stating TOF count 1 or 2 as appropriate for intubation. Arguments could be made for and against both approaches: Some variability in the onset of neuromuscular blockers must be expected in all patients, especially those with organ disease and with varying doses.^16^, ^17^ On the other hand, the appropriateness of relying on neuromuscular monitoring would also depend on at what TOF value one chooses to perform intubation, as peak relaxation at the larynx and mandible precedes that at the adductor pollicis.^18^ The type and dosage of hypnotics and opioids also influences intubation conditions.^18^ We suggest to use objective (and automatic) neuromuscular monitoring with stimulation every 12th second *combined* with a timer. Laryngoscopy may then be initiated after at least 1.5 minutes and a TOF count of 1 or 0, likely resulting in intubation at TOF count 0. This is debatable.

The US residents used subjective neuromuscular monitoring and clinical tests to assess for readiness for extubation or need for reversal. The limitations of subjective neuromuscular monitoring to detect residual block has been established and it has therefore been suggested to administer at least a small dose of neostigmine to all patients.^19^, ^20^ However, not all residents were aware of this approach. The clinical tests are also unreliable in assessing readiness for extubation.^21^ Finally, the attendings use varying approaches, often stating time since last dosage of NMBA as a reference for need for reversal. It is therefore understandable that residents rely on heuristics like ‘time since last dosage of NMBA’ to decide whether to administer reversal, as also described in a Brazilian study, though this is not optimal practice.^22^ There is, however, a large variability in the duration of action of the non‐depolarizing NMBAs with intermediate action, as more than a third of patients will have a TOF ratio <0.9 two hours after a standard intubation dose of two times the ED_95_.^23^ Most Danish anaesthetists would monitor the TOF ratio continuously and omit reversal as appropriate. When necessary, they would use a standard non‐weight‐based dose of neostigmine and follow the TOF response on the monitoring screen, re‐dosing reversal if needed until a TOF ratio >0.9 was achieved. For the average patient weighing 70 kg, this neostigmine dosing corresponds to 35 µg/kg, which is close to the 30 µg recommended as a minimal dose when objective neuromuscular monitoring is not available.^20^ Since the response to the standardized dose is directly observed with TOF measurements typically every 12 seconds, re‐dosing of reversal or extubation can be performed as appropriate. The residents reported a varying dosing of neostigmine, with some routinely administering at least a small dose as recommended under the circumstances.^20^ While this small dose is unlikely to cause serious side effects, it is worth noting that neostigmine can be omitted completely when objective neuromuscular monitoring documents complete recovery. This difference in neostigmine dosing habits depending on equipment availability was also demonstrated in a survey of US and European anaesthesiologists.^10^

### 4.2 Barriers and aids to routine neuromuscular monitoring

For four decades, there has been a strong tradition for research and training in neuromuscular monitoring in Denmark, and The Danish Anaesthesia Society recommends that objective neuromuscular monitoring should be used with non‐depolarizing NMBAs and considered with succinylcholine.^24^ The American Society of Anesthesiologists’ standards for anaesthetic monitoring, on the other hand, do not mention neuromuscular monitoring,^2^ whereas their guideline for post‐anaesthetic care from 2013 only mentions that ‘Assessment of neuromuscular function primarily includes physical examination and, on occasion, may include neuromuscular blockade monitoring’.^25^ It is not enough, however, just to make objective neuromuscular monitoring available and mandatory.^2^ We found that the attitude of both the residents and the attendings influenced the use of neuromuscular monitoring, with many of the residents not appreciating a potential benefit of objective neuromuscular monitoring over subjective. For department culture to change, training in neuromuscular monitoring should be consistent and lead by clinicians with experience using the equipment, and surveys of residual neuromuscular block should be conducted locally to increase awareness.^2^, ^8^, ^9^ The interviews with Danish anaesthetists also revealed a need for further training in the purpose of calibration of the objective neuromuscular monitoring device and dealing with TOF ratios >1.0, as was also described in a recent Danish survey.^12^ A challenge identified was placing the monitor when the patient's arms were tucked to the sides. Electromyography, as opposed to acceleromyography, could potentially eliminate this challenge because it does not require a freely moving thumb.^2^ A further advantage of electromyography would be a control TOF ratio closer to 1.0.^26^ We found that even when objective neuromuscular monitoring was available, the residents at the US institution were reluctant to use it, either from lack of training or because they did not find it necessary. In a survey from 2010, US respondents were also less likely to use objective neuromuscular monitoring than European respondents, even if both subjective and objective monitoring were available.^10^

Both in Denmark and the US institution, anaesthetists agreed that residual neuromuscular block is a serious complication and that it probably occurs more frequently than experienced. This in concordance with surveys of anaesthesiologists’ opinions and attitude about residual neuromuscular block, where the incidence is often underestimated and thought to be higher in colleagues’ practices.^6^, ^11^, ^22^ In a recent study assessing the incidence of residual neuromuscular block in 10 US hospitals, including the institution studied, 65% of patients receiving a non‐depolarizing NMBA had a TOF ratio <0.9 at extubation.^27^ Though our study was not quantitatively designed, it is worth noticing that several of the residents at the US institution had experienced cases with severe residual neuromuscular block, including need for re‐intubation, already within the first years of training, whereas in Denmark, only senior consultants reported that type of experience.

### 4.3 Neuromuscular monitoring after succinylcholine

Although patients with undiagnosed BChE deficiency are at high risk of experiencing awareness during emergence if neuromuscular monitoring is not applied, neither Danish anaesthetists nor the residents monitored the depolarizing block routinely.^3^ Both Danish and US anaesthetists reported senior colleagues who would not acknowledge residual neuromuscular block due to BChE deficiency, as was also described in a study of cases from the Danish BChE registry.^3^

### 4.4 Strengths and limitations

The Danish part of the study included focus group interviews with both nurse anaesthetists and anaesthesiologists with a range of experience, from hospitals of varying size, resulting in a reasonably representative sample of the 30 public anaesthesia departments in Denmark. On the other hand, findings from our sample of three groups of US anaesthesia residents is not representative of anaesthesia practice across the US, but may still provide relevant input to the process of implementation of objective neuromuscular monitoring. The interviews in Denmark and the US were conducted three years apart. If and how this might have affected our findings remains unknown.

### 4.5 Implications

While almost fully implemented in Denmark, objective neuromuscular monitoring still poses challenges in daily clinical practice. Training is needed in troubleshooting the potential pitfalls that exist with these devices which are not described in standard anaesthesia textbooks. Continuing medical education in this topic could include e‐learning modules that can easily be disseminated to a larger audience.^28^ In US institutions that aim to implement objective neuromuscular monitoring, residents should be motivated to use the equipment not only by the reduction of residual neuromuscular block and postoperative pulmonary complications, but also the potential to simplify assessment of need for reversal and readiness for extubation. There is still a need for a culture change, also among senior anaesthesiologists, putting an end to the incorrect belief that they can rely on subjective neuromuscular monitoring to rule out residual neuromuscular block.^29^ The US institution studied is in the process of implementing objective neuromuscular monitoring, bearing the described barriers in mind, and has developed a CME accredited e‐learning module about objective neuromuscular monitoring and residual neuromuscular blockade.

## 5 CONCLUSION

Using qualitative methods, we found that though Danish anaesthetists generally apply objective neuromuscular monitoring routinely and residents at the US institution often apply subjective neuromuscular monitoring, barriers to consistent and correct use still exist. They include unreliable equipment, time pressure, need for training, misconceptions about NMBA pharmacokinetics and residual block, lack of standards and guidelines and departmental culture.

## CONFLICT OF INTEREST

JLDT: Received grants and speaker's fees from Merck. APM: None declared. SW: None declared. AM: None declared. PT: None declared. MRG: Received grants and speaker's fees from Merck. DØ: None declared.

## AUTHOR CONTRIBUTIONS

JLDT: Design of the study, data collection, analysis and interpretation of data and drafting the manuscript. APM, MRG and DØ: Design of the study, data collection, interpretation of data and revising the manuscript. SW, PT: Data collection, interpretation of data and revising the manuscript. AM: Data collection and revising the manuscript. All authors approved the final version and agreed to be accountable for all aspects of the work.

## Supporting information

Appendices S1‐S2

## References

[1] Brull SJ , Murphy GS . Residual neuromuscular block: lessons unlearned. Part II: methods to reduce the risk of residual weakness. Anesth Analg. 2010;111:129‐140. 20442261 10.1213/ANE.0b013e3181da8312

[2] Naguib M , Brull SJ , Kopman AF , et al. Consensus statement on perioperative use of neuromuscular monitoring. Anesth Analg. 2018;127:71‐80. 29200077 10.1213/ANE.0000000000002670

[3] Thomsen JL , Nielsen CV , Eskildsen KZ , Demant MN , Gätke MR . Awareness during emergence from anaesthesia: significance of neuromuscular monitoring in patients with butyrylcholinesterase deficiency. Br J Anaesth. 2015;115(Suppl):i78‐i88. 26174305 10.1093/bja/aev096

[4] Aytac I , Postaci A , Aytac B , et al. Survey of postoperative residual curarization, acute respiratory events and approach of anesthesiologists. Braz J Anesthesiol. 2016;66:55‐62. 26768931 10.1016/j.bjane.2012.06.011

[5] Chacko C , Haldar M . Survey on neuromuscular management. J Anaesthesiol Clin Pharmacol. 2016;32:122. 27006561 10.4103/0970-9185.173366 PMC4784197

[6] Phillips S , Stewart P , Bilgin A . A survey of the management of neuromuscular blockade monitoring in Australia and New Zealand. Anaesth Intensive Care. 2013;41:374‐379. 23659401 10.1177/0310057X1304100316

[7] Baillard C , Clec'h C , Catineau J , et al. Postoperative residual neuromuscular block: a survey of management. Br J Anaesth. 2005;95:622‐626. 16183681 10.1093/bja/aei240

[8] Todd MM , Hindman BJ , King BJ . The implementation of quantitative electromyographic neuromuscular monitoring in an academic anesthesia department. Anesth Analg. 2014;119:323‐331. 24878683 10.1213/ANE.0000000000000261

[9] Todd MM , Hindman BJ . The implementation of quantitative electromyographic neuromuscular monitoring in an academic anesthesia department: follow‐up observations. Anesth Analg. 2015;121:836‐838. 10.1213/ANE.0000000000000760 26287308

[10] Naguib M , Kopman AF , Lien CA , Hunter JM , Lopez A , Brull SJ . A survey of current management of neuromuscular block in the United States and Europe. Anesth Analg. 2010;111:110‐119. 19910616 10.1213/ANE.0b013e3181c07428

[11] Sorgenfrei IF , Viby‐Mogensen J , Swiatek FA . Does evidence lead to a change in clinical practice? Danish anaesthetists’ and nurse anesthetists’ clinical practice and knowledge of postoperative residual curarization. Ugeskr Laeger. 2005;167:3878‐3882. 16221428

[12] Söderström CM , Eskildsen KZ , Gätke MR , Staehr‐Rye AK . Objective neuromuscular monitoring of neuromuscular blockade in Denmark: an online‐based survey of current practice. Acta Anaesthesiol Scand. 2017;61:619‐626. 28573656 10.1111/aas.12907

[13] Malterud K . Kvalitative metoder i medisinsk forskning. Oslo, Norway: Universitetsforlaget; 2006.

[14] Tong A , Sainsbury P , Craig J . Consolidated criteria for reporting qualitative research (COREQ): a 32‐item checklist for interviews and focus groups. Int J Qual Health Care. 2007;19:349‐357. 17872937 10.1093/intqhc/mzm042

[15] Torre DM , Daley BJ , Sebastian JL , Elnicki DM . Overview of current learning theories for medical educators. Am J Med. 2006;119:903‐907. 17000227 10.1016/j.amjmed.2006.06.037

[16] Craig RG , Hunter JM . Neuromuscular blocking drugs and their antagonists in patients with organ disease. Anaesthesia. 2009;64(Suppl 1):55‐65. 19222432 10.1111/j.1365-2044.2008.05871.x

[17] Schultz P , Ibsen M , Østergaard D , Skovgaard LT . Onset and duration of action of rocuronium–from tracheal intubation, through intense block to complete recovery. Acta Anaesthesiol Scand. 2001;45:612‐617. 11309014 10.1034/j.1399-6576.2001.045005612.x

[18] Kopman AF , Klewicka MM , Neuman GG . Reexamined: the recommended endotracheal intubating dose for nondepolarizing neuromuscular blockers of rapid onset. Anesth Analg. 2001;93:954‐959. 11574363 10.1097/00000539-200110000-00030

[19] Viby‐Mogensen J , Jensen NH , Engbaek J , Ording H , Skovgaard LT , Chraemmer‐Jørgensen B . Tactile and visual evaluation of the response to train‐of‐four nerve stimulation. Anesthesiology. 1985;63:440‐443. 4037404 10.1097/00000542-198510000-00015

[20] Murphy GS , Kopman AF . “To reverse or not to reverse?”. Anesthesiology. 2016;125(4):611‐614. 27496655 10.1097/ALN.0000000000001280

[21] Cammu G , De Witte J , De Veylder J , et al. Postoperative residual paralysis in outpatients versus inpatients. Anesth Analg. 2006;102:426‐429. 16428537 10.1213/01.ane.0000195543.61123.1f

[22] Videira RLR , Vieira JE . What rules of thumb do clinicians use to decide whether to antagonize nondepolarizing neuromuscular blocking drugs? Anesth Analg. 2011;113:1192‐1196. 21865491 10.1213/ANE.0b013e31822c986e

[23] Debaene B , Plaud B , Dilly M , Donati F , Ph D . Residual paralysis in the PACU after a single intubating dose of nondepolarizing muscle relaxant with an intermediate duration of action. Anesthesiology. 2003;98:1042‐1048. 12717123 10.1097/00000542-200305000-00004

[24] Viby‐Mogensen J , Jørgensen BC , Ording H . Residual curarization in the recovery room. Anesthesiology. 1979;50:539‐541. 156513 10.1097/00000542-197906000-00014

[25] Apfelbaum JL , Silverstein JH , Chung FF , et al. Practice guidelines for postanesthetic care: an updated report by the American Society of Anesthesiologists Task Force on Postanesthetic Care. Anesthesiology. 2013;118:291‐307. 23364567 10.1097/ALN.0b013e31827773e9

[26] Kopman AF , Chin W , Cyriac J . Acceleromyography vs. electromyography: an ipsilateral comparison of the indirectly evoked neuromuscular response to train‐of‐four stimulation. Acta Anaesthesiol Scand. 2005;49:316‐322. 15752395 10.1111/j.1399-6576.2005.00643.x

[27] Saager L , Maiese EM , Bash LD , et al. Incidence, risk factors, and consequences of residual neuromuscular block in the United States: The prospective, observational, multicenter RECITE‐US study. J Clin Anesth. 2019;55:33‐41. 30594097 10.1016/j.jclinane.2018.12.042

[28] Thomsen JLD , Mathiesen O , Hägi‐Pedersen D , et al. Improving neuromuscular monitoring and reducing residual neuromuscular blockade with e‐learning: protocol for the multicenter interrupted time series INVERT study. JMIR Res Protoc. 2017;6:e192. 28986337 10.2196/resprot.7527 PMC5650673

[29] Naguib M , Brull SJ , Hunter JM , Kopman AF , Fülesdi B , Johnson KB . Arkes HR. Anesthesiologists’ overconfidence in their perceived knowledge of neuromuscular monitoring and its relevance to all aspects of medical practice: an international survey. Anesth Analg. 2019;128:1118‐1126. 31094776 10.1213/ANE.0000000000003714

