# Supplementary material for: Barriers and aids to routine neuromuscular monitoring and consistent reversal practice—A qualitative study
Source: Acta Anaesthesiol Scand. 2020 May 6;64(8):1089–99. doi: 10.1111/aas.13606 (PMC7497053; doi:10.1111/aas.13606)
Supplement: Supplementary file 1 — Appendices S1‐S2 [file AAS-64-1089-s001.docx]

# Appendix 1

**Interview guide: focus group interview**

| **Theme** | **Questions** |
| --- | --- |
| **Neuromuscular monitoring** | - What types of neuromuscular monitoring equipment is available in the operating rooms? - When do you apply neuromuscular monitoring? - Before administering an NMBA?? - Do you apply neuromuscular monitoring when administering succinylcholine only? - Do you use other stimulation patterns than train-of-four? Which? When? - Do you experience challenges in interpreting the response? - Challenges in detecting fade in non-depolarizing neuromuscular block? - Does the equipment you use ever fail? - Have you ever used an objective/quantitative neuromuscular monitor? - Do you note the TOF measurements in the electronic medical record? |
| **Residual blockade** | - Can you describe what you typically do before extubating? - When is it safe to extubate the patient? What criteria? - How do you use neuromuscular monitoring in relation to extubation? - Do you use clinical tests to assess for residual blockade? Which? - Please discuss the specificity and/or sensitivity of the clinical tests? - Have you experienced patients with residual blockade? Please describe. - Does residual blockade affect patient safety? - Have you experienced patients with pseudocholinesterase deficiency? |
| **Reversal** | - When do you administer reversal? Who decides? - When do you **not** administer reversal? At what TOF count? - What dose do you use? Is there a guideline or similar for dosing? - Do you use the same dose for everyone? - Do you give repeated doses? when? - Do you apply neuromuscular monitoring after reversal? |
| **Needs** | - What do you think influences your behavior in relation to relaxants, monitoring, reversal? - What areas of neuromuscular transmission, monitoring, and reversal would you like to know more about? - What do you think it takes to make you and your colleagues change practice? |

# Appendix 2: Use of neuromuscular monitoring and reversal

Themes, subthemes, codes, and illustrative quotes from focus group interviews and surveys in Denmark (DK) and the institution in the United States (US). The number after ‘DK’ indicates department number, while the number after ‘US’ indicates year of residency for the interviewee. ‘S’ indicates data from surveys. TOF: Train-of-four, NMBA: neuromuscular blocking agent, BChE: butyrylcholinesterase

| **Subtheme** | **Code** | **Source** | **Quote or observation** |
| --- | --- | --- | --- |
| **Baseline** | Calibration | DK-1 | If I anticipate that rocuronium will be required, I put the TOF monitor on before administering it to have a baseline measurement |
|  |  | US-1 | [Attending] at the [other hospital] is the only person that I have done that with – you will give propofol and they’ll be unconscious and before you give the paralytic, twitch to see what their full strength looks like before. |
| **Intubation** | Guide intubation | DK-3 | We start looking [with the laryngoscope] when it says TOF 1 or 2 |
|  | Guide intubation | US (S) | I use it for induction to assess effect prior to intubation, and again, periodically throughout |
|  | Time from NMBA to intubation | US-2 | Usually two minutes, if everyone’s willing to wait  I set a timer, there is a timer on the machine, and then I wait two minutes.  Most attendings don’t want to wait. |
| **Maintenance** | Check manually | US-2 | I’ll just have it on and check every 30, 20 minutes depending on how much block the patient’s needed previously. |
| **Assessing need for reversal** | Clinical tests | US-3 | But yeah, I think if like they’ve moved or if I have like switched them over to like manual and they’re breathing on their own, taking big tidal volumes, sometimes I wouldn’t reverse them, you know. And especially if I wasn’t like re-dosing the relaxant throughout the case and they just had it at the beginning for intubation. |
|  | Clinical tests/  Time since NMBA | US (S) | Ensure spontaneous ventilation, adequate time after NM blocker |
|  | Routine obj NM | DK-5 | You watch the TOF ratio return and keep them sedated, and if you can time it by reducing or terminating the infusion, then when the TOF ratio reaches 80-90%, they will be awake and alert, and of course they have to be able to breathe normally. |
|  | Decision based on objective NM | DK-5 | If I am ready to awaken them, and they have not reached the percentages, but are staying down at 2-3 TOF counts, then I give reversal, but if they are at 35-40% and I can follow them all the way to 80-90%, then I do not reverse. |
|  | Time since NMBA | US-1 | I’ve been taught by many attendings that if it’s a young healthy patient with no other like pulmonary or cardiac comorbidities and the case is greater than 2 hours and they have full twitches that you don’t have to reverse, if they only received one intubating dose of rocuronium or non-depolarizing. |
|  | Time since NMBA | US (S) | [I do not administer reversal after a] single dose of muscle relaxant, at the start of a long (>2 hour ) case |
|  | Time since NMBA | US-2 | Some certain attendings have kind of not wanted to reverse if it’s been sufficiently long and there is obviously **subjective** full strength |
|  | Time since NMBA | US (S) | Reverse any person who has received a dose of non-depolarizer within 6 hrs |
|  | Clinical signs | US-3 | But I don’t always check before I reverse. (…) If they moved or if they’ve shown you that they’re not fully paralyzed. Then I feel comfortable enough reversing. |
|  | Always give reversal | US-1 | That’s why I always give 1 and 0.2, and some of the old school attendings say ‘what is this [low] dosing?’, I’m like ‘well it’s not full reversal but it’s a little something just to…’ It’s called a “legal reversal” – or a “CYA reversal” |
|  | Risk/benefit | US-2 | [I assess the] risk/benefit of giving neostigmine, is this an older patient and worried about urinary retention or is this like a young woman where I’m worried about PONV, like if it’s been four hours I don’t really want to give neostigmine to them. |
| **Neostigmine dosing** | Based on subjective NM | US-2 | If they have four reasonably strong looking twitches and it’s been longer than an hour, I’ll just give 20 mics per of you know neostigmine and if they have definitely fewer than four twitches, I’ll give 50. |
|  | Fixed dose, weight based | US-3 | I tend to just give the same dose actually because of what I heard about people having residual block in the PACU. |
|  | Fixed dose, non-weight based | DK-2 | We give 1 ml and then follow the TOF response |
| **Neostigmine effect** | Limitations of subjective NM | US-2 | I take it off as soon as I have determined how many twitches they have (...) because I presume that they are going to go to four twitches and I won’t be able to assess the strength visually anyway. |
|  | Tetanus | US-1 | Usually I will do TOF and then I will do tetanus |
|  | Clinical tests | US-1 | I check twitches, give however much reversal based on that and switch them over to pressure support manual and then see what their tidal volumes are and then take the tube once it kind of looks satisfactory. |
|  | Limitations of clinical tests | DK-5 | There are limitations to what tests you can use on the operating table before you extubate. It could be a head lift, because all the other tests, biting a tongue depressor, hand squeeze and coordination, we cannot use those because the patient is still groggy from anesthesia, so you will not get a valid result. |
